# Supplementary material for: Impact of Biologics on Comorbidities in Patients with Psoriasis or Psoriatic Arthritis
Source: Biomedicines. 2025 Sep 10;13(9):2219. doi: 10.3390/biomedicines13092219 (PMC12466972; doi:10.3390/biomedicines13092219)
Supplement: Supplementary file 1 [file biomedicines-13-02219-s001.zip › biomedicines-3813705-Supplementary materials_final.pdf]

Supplementary materials

Impact of biologics on comorbidities in patients with psoriasis or psoriatic arthritis

Biomedicines

S.-H.L.; S.L.; H.S.S.; S.B.K.; M.E.; S.-P.H.

Table S1. Definitions of outcome measures and comorbid diseases

Table S2. Baseline characteristics of the entire study population

Table S3. Characteristics of the general health examination subgroup

References for supplementary tables

Figure S1. Cumulative incidences of comorbid diseases outcomes associated with biologics

Figure S2. Head-to-head comparison of the risks of comorbid diseases associated with biologics across class of IL inhibitors

This supplementary material has been provided by the authors to give readers additional information about their work.

Table S1. Definitions of outcome measures and comorbid diseases

**Definitions of outcome measures**	
The primary outcome of interest was the prevalence and incidence of comorbid diseases associated with biologics identified by at least three documented visits with the corresponding ICD-10 code for each disease until December 31, 2021. MACE was defined as having at least one hospitalization with myocardial infarction or stroke as the principal diagnosis	
**Primary outcome of interest**	**ICD-10 code**
**Cardiovascular diseases**	
MACE	I21, I24, I60, I61, I62, and I63
Myocardial infarction	I21 and I24
Stroke	I60, I61, I62, and I63
Congestive heart failure	I50
Atrial fibrillation	I48
Transient ischaemic attack	G45
Peripheral arterial disease	I73
Atherosclerosis	I70
**Autoimmune/inflammatory diseases**	
Rheumatoid arthritis	M05, M06, and M08
Inflammatory bowel disease	K50 and K51
Vasculopathy	M30 and M31
Immunobullous disease	L10, L100, L102, L109, L123, and L120
**Infectious diseases**	
Tuberculosis	A15, A17, A18, and A19
Hepatitis B virus infection	B180, B181, and B191
Hepatitis C virus infection	B182 and B192
Human immunodeficiency virus infection	B20
Herpes zoster	B02
**Psychiatric diseases**	
Mood disorder	F32 and F33
Anxiety disorder	F4
Dementia	G30, G31, F01, F02, and F03
**Malignancies**	
Lymphoma	C90–95
Leukaemia	C81–88
Solid tumour	C0–7
Non-melanoma skin cancer	C44
Malignant melanoma	C43
**Other diseases**	
Asthma	J45
Chronic obstructive pulmonary disease	J44
Chronic kidney disease	N18

ICD-10, International Statistical Classification of Diseases, Tenth Revision; MACE, major adverse cardiovascular event

Table S2. Baseline characteristics of the entire study population

Characteristics	Cases, No. (%) Biologics (n = 8,173)		Controls (n = 41,598)		*P*-value
Psoriatic arthritis	971	(11.9%)	115	(0.3%)	<0.001
Age, mean (SD), y	49.1	(14.1)	52.7	(16.7)	<0.001
Follow-up duration, mean (SD), y	3.8	(3.0)	7.3	(4.7)	<0.001
Sex					
Male	5,413	(66.2%)	23,944	(57.6%)	<0.001
Female	2,760	(33.8%)	17,654	(42.4%)	
Insurance type					
Standard	7,425	(90.9%)	38,158	(92.7%)	0.009
Medicaid	748	(9.1%)	3,440	(7.3%)	
Income level quartile					
Highest	2,532	(31.0%)	11,520	(27.7%)	<0.001
Higher	2,624	(32.1%)	12,995	(31.2%)	
Lower	2,017	(24.7%)	11,483	(27.6%)	
Lowest	1,000	(12.2%)	5,600	(13.5%)	
Type of location					
Metropolitan	3,793	(46.4%)	20,669	(49.7%)	<0.001
Other area	4,380	(53.6%)	20,929	(50.3%)	
Class of biologics					
TNF-α inhibitor	2,069	(25.3%)	-		N/A
IL-12/23 inhibitor	2,170	(26.6%)	-		N/A
IL-23 inhibitor	2,437	(29.8%)	-		N/A
IL-17 inhibitor	1,497	(18.3%)	-		N/A
Past medical history					
Hypertension	1,121	(13.7%)	5,356	(12.9%)	0.041
Diabetes	453	(5.5%)	2,411	(5.8%)	0.383
Hyperlipidaemia	289	(3.5%)	1,482	(3.6%)	0.931

AIDS, acquired immune deficiency syndrome; HBV, hepatitis B virus; HCV, hepatitis C virus; N/A, not applicable; No., number; IL, interleukin; PsO, psoriasis; SD, standard deviation; TNF, tumour necrosis factor.

Table S2 summarises the baseline characteristics of the biologics and control cohorts. The biologics cohort had a significantly higher prevalence of psoriatic arthritis (11.9% vs 0.3%; $p < .001$), likely reflecting the preferential use of biologics for managing both skin and joint symptoms in PsO. This cohort was also younger (mean age 49.1 vs. 52.7 years; $p < .001$) and had a shorter mean follow-up duration (3.8 vs. 7.3 years; $p < .001$), likely attributable to the relatively recent introduction of biologics compared with conventional systemic immunosuppressants. However, the shorter follow-up period may limit the ability to fully assess long-term comorbidities in this cohort.

The sex distribution revealed a higher proportion of male patients in the biologics cohort (66.2% vs 57.6%; $p < .001$), potentially reflecting greater disease severity in males.[43,44] Socioeconomic factors, such as income level, also differed significantly, with a higher proportion of patients in the biologics cohort in the highest income quartile (31.0% vs 27.7%; $p < .001$). These differences may reflect disparities in treatment accessibility rather than direct effects on disease outcomes.

Although certain variables, such as hypertension (13.7% vs 12.9%; $p = .041$), reached statistical significance, their small absolute differences are unlikely to be clinically meaningful. Notable differences, including the higher prevalence of psoriatic arthritis and the male predominance in the biologics cohort, may contribute to the observed differences in outcomes.

Table S3. Characteristics of the general health examination subgroup

Characteristics	Cases, No. (%)				*P*-value
	Biologics (n = 6,932)		Controls (n = 35,096)		
Psoriatic arthritis	833	(12.0%)	98	(0.3%)	<0.001
Age, mean (SD), y	50.9	(13.2)	54.8	(15.3)	<0.001
Follow-up duration, mean (SD), y	3.8	(3.0)	7.5	(4.4)	<0.001
Sex					
Male	4,601	(66.4%)	20,229	(57.6%)	<0.001
Female	2,331	(33.6%)	14,867	(42.4%)	
Insurance type					
Standard	6,354	(91.7%)	32,531	(92.7%)	<0.001
Medicaid	578	(8.3%)	2,565	(7.3%)	
Income level					
Highest	2,141	(30.9%)	9,759	(27.8%)	<0.001
Higher	2,291	(33.0%)	11,227	(32.0%)	
Lower	1,696	(24.5%)	9,608	(27.4%)	
Lowest	804	(11.6%)	4,502	(12.8%)	
Type of location					
Metropolitan	3,160	(45.6%)	17,370	(49.5%)	<0.001
Other area	3,772	(54.4%)	17,726	(50.5%)	
Class of biologics					
TNF-α inhibitor	1,722	(24.8%)	-		N/A
IL-12/23 inhibitor	1,864	(26.9%)	-		N/A
IL-23 inhibitor	2,084	(30.1%)	-		N/A
IL-17 inhibitor	1,262	(18.2%)	-		N/A
Past medical history					
Hypertension	1,042	(15.0%)	4,934	(14.1%)	0.036
Diabetes	411	(5.9%)	2,182	(6.2%)	0.377
Dyslipidaemia	278	(4.0%)	1,408	(4.0%)	1.000
Tobacco use	1,912	(27.6%)	8,574	(24.4%)	<0.001
Obesity	3,444	(49.7%)	14,715	(41.9%)	<0.001
BMI, mean (SD), kg/m^2	25.3	(4.1)	24.6	(3.7)	<0.001
No. of CV risk factors					
0	1,447	(20.9%)	7,820	(22.3%)	0.010
1	2,392	(34.5%)	11,685	(33.3%)	0.052
2	1,829	(26.4%)	9,556	(27.2%)	0.153
3	981	(14.2%)	4,750	(13.5%)	0.177
4	266	(3.8%)	1,174	(3.4%)	0.043
5	16	(0.2%)	109	(0.3%)	0.320
6	1	(0.0%)	2	(0.0%)	0.994

BMI, body mass index; CV, cardiovascular; N/A, not applicable; No., number; IL, interleukin; PsO, psoriasis; SD, standard deviation; TNF, tumour necrosis factor

Table S3 presents additional baseline characteristics of the subgroup undergoing general health examinations in the biologics and control cohorts. Notable differences include a higher prevalence of tobacco use in the

biologics cohort (27.6% vs 24.4%; $p < .001$), potentially reflecting the association between smoking and severe PsO.[45,46] Obesity was also more prevalent in the biologics cohort (49.7% vs 41.9%; $p < .001$), accompanied by a higher mean BMI (25.3 vs 24.6 kg/m^2; $p < .001$), consistent with the established links between obesity, systemic inflammation, and PsO severity.[47-49]

Slight differences were observed in the distribution of CV risk factors, with the biologics cohort showing a marginally lower proportion of patients with zero CV risk factors (20.9% vs 22.3%; $p = .010$) and a slightly higher proportion with four CV risk factors (3.8% vs 3.4%; $p = .043$). While these differences suggest a slightly greater comorbid burden in the biologics cohort,[50,51] the absolute differences are small and should be interpreted cautiously. These findings emphasise the complex interplay among PsO severity, lifestyle factors, and systemic comorbidities, underscoring the need for comprehensive management strategies that address both disease severity and modifiable risk factors.

References for Supplementary Tables

43. Guillet, C.; Seeli, C.; Nina, M.; Maul, L.V.; Maul, J.T. The impact of gender and sex in psoriasis: What to be aware of when treating women with psoriasis. *Int J Womens Dermatol* **2022**, *8*, e010, doi:10.1097/jw9.0000000000000010.
44. Hägg, D.; Sundström, A.; Eriksson, M.; Schmitt-Egenolf, M. Severity of Psoriasis Differs Between Men and Women: A Study of the Clinical Outcome Measure Psoriasis Area and Severity Index (PASI) in 5438 Swedish Register Patients. *Am J Clin Dermatol* **2017**, *18*, 583-590, doi:10.1007/s40257-017-0274-0.
45. Armstrong, A.W.; Harskamp, C.T.; Dhillon, J.S.; Armstrong, E.J. Psoriasis and smoking: a systematic review and meta-analysis. *Br J Dermatol* **2014**, *170*, 304-314, doi:10.1111/bjd.12670.
46. Armstrong, A.W.; Armstrong, E.J.; Fuller, E.N.; Sockolov, M.E.; Voyles, S.V. Smoking and pathogenesis of psoriasis: a review of oxidative, inflammatory and genetic mechanisms. *Br J Dermatol* **2011**, *165*, 1162-1168, doi:10.1111/j.1365-2133.2011.10526.x.
47. Budu-Aggrey, A.; Brumpton, B.; Tyrrell, J.; Watkins, S.; Modalsli, E.H.; Celis-Morales, C.; Ferguson, L.D.; Vie, G.; Palmer, T.; Fritsche, L.G.*, et al.* Evidence of a causal relationship between body mass index and psoriasis: A mendelian randomization study. *PLoS Med* **2019**, *16*, e1002739, doi:10.1371/journal.pmed.1002739.
48. Kunz, M.; Simon, J.C.; Saalbach, A. Psoriasis: Obesity and Fatty Acids. *Front Immunol* **2019**, *10*, 1807, doi:10.3389/fimmu.2019.01807.
49. Chiricozzi, A.; Raimondo, A.; Lembo, S.; Fausti, F.; Dini, V.; Costanzo, A.; Monfrecola, G.; Balato, N.; Ayala, F.; Romanelli, M.*, et al.* Crosstalk between skin inflammation and adipose tissue-derived products: pathogenic evidence linking psoriasis to increased adiposity. *Expert Rev Clin Immunol* **2016**, *12*, 1299-1308, doi:10.1080/1744666x.2016.1201423.
50. Gao, N.; Kong, M.; Li, X.; Zhu, X.; Wei, D.; Ni, M.; Wang, Y.; Hong, Z.; Dong, A. The Association Between Psoriasis and Risk of Cardiovascular Disease: A Mendelian Randomization Analysis. *Front Immunol* **2022**, *13*, 918224, doi:10.3389/fimmu.2022.918224.
51. Armstrong, E.J.; Harskamp, C.T.; Armstrong, A.W. Psoriasis and major adverse cardiovascular events: a systematic review and meta-analysis of observational studies. *J Am Heart Assoc* **2013**, *2*, e000062, doi:10.1161/jaha.113.000062.

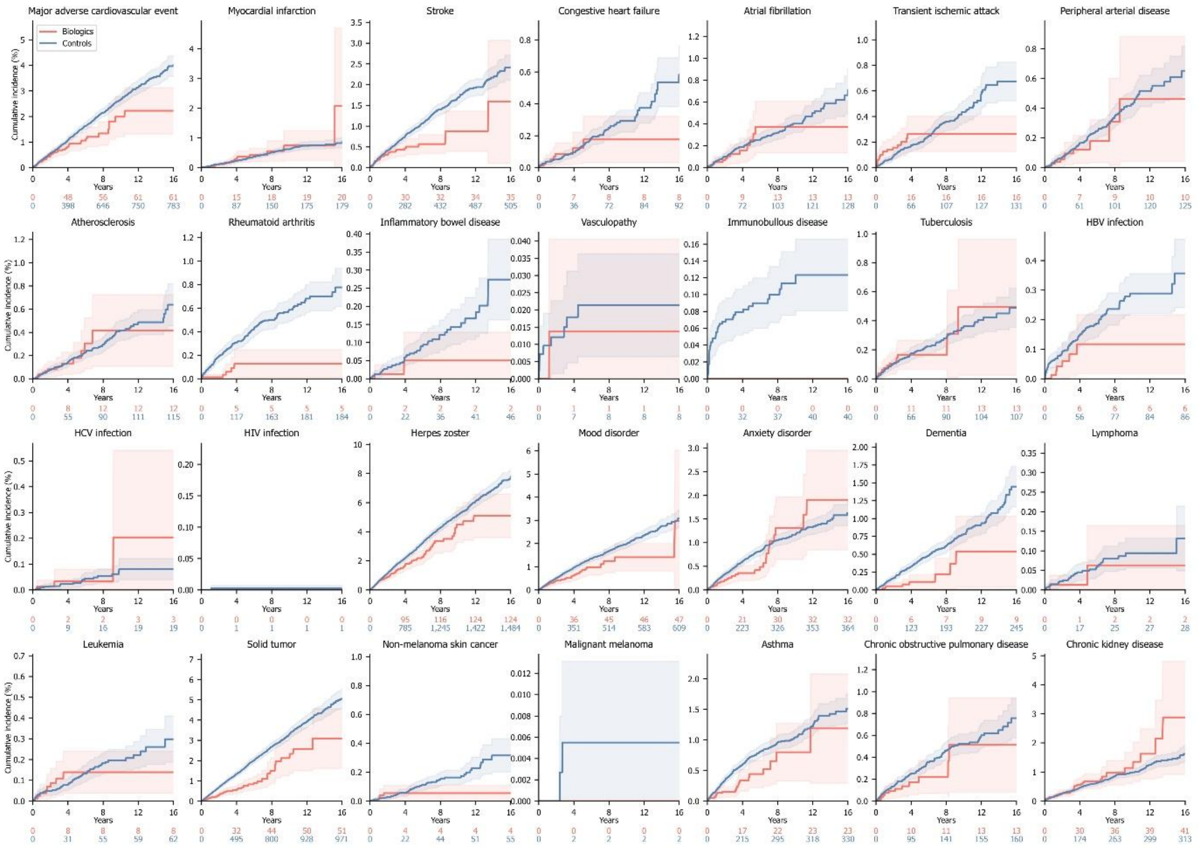

Figure S1. Cumulative incidences of comorbid diseases outcomes associated with biologics The cumulative incidence plot shows the cumulative incidence functions and the number of comorbid diseases in biologics cohort and control cohort. The shaded area shows the 95% confidence interval for the cumulative incidence.

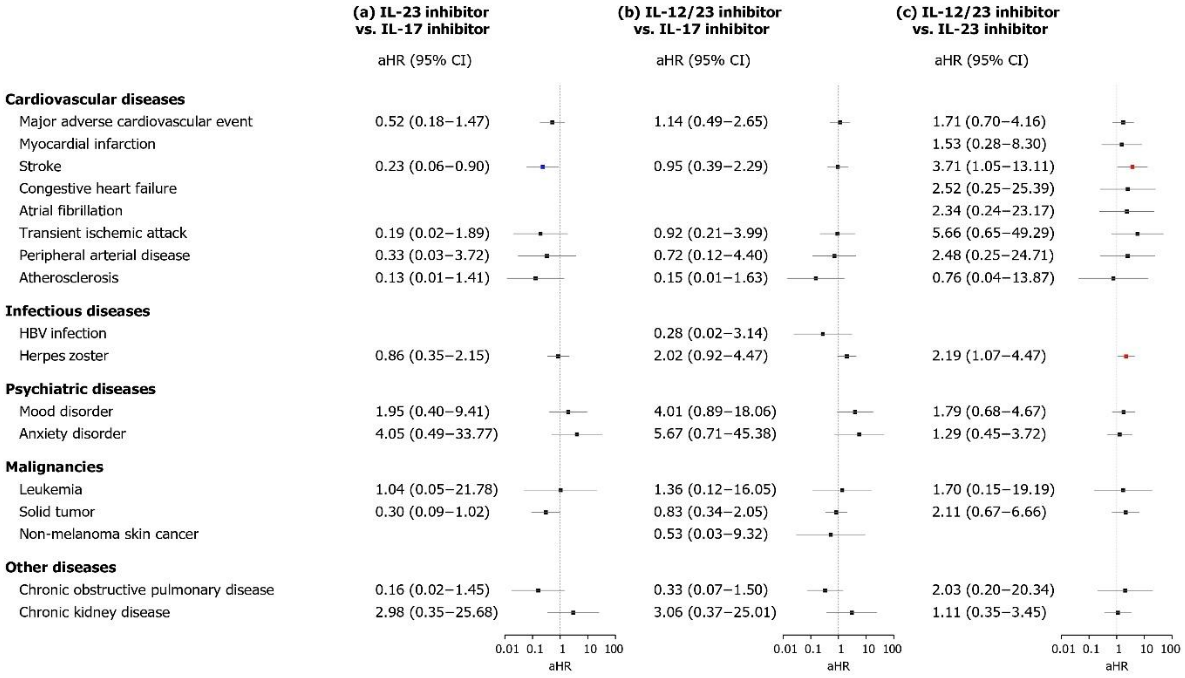

Figure S2. Head-to-head comparison of the risks of comorbid diseases associated with biologics across class of IL inhibitors

The forest plot illustrates the adjusted hazard ratios (aHRs) and 95% confidence intervals (CIs) for comorbid diseases across class of IL inhibitors, including IL-12/23 inhibitor, IL-23 inhibitor, and IL-17 inhibitor. Statistical estimates were adjusted for age, sex, insurance type, income level, and location. The risks of comorbid disease outcomes were compared directly between class of biologics: (a) IL-23 inhibitor vs. IL-17 inhibitor, (b) IL-12/23 inhibitor vs. IL-17 inhibitor, and (c) IL-12/23 inhibitor vs. IL-23 inhibitor. aHRs, adjusted hazard ratios; CI, confidence interval; IL, interleukin
